# The FSIP Family: Roles in Health and Cancer

**DOI:** 10.3390/cancers17193107

**Published:** 2025-09-24

**Authors:** Zhan Zhang, Yunfan Liu, Chao Liu, Lujia Qin, Mone Zaidi, Caigang Liu

**Affiliations:** 1 Department of Oncology, Shengjing Hospital of China Medical University, Shenyang 110022, China; zhangzhan@cmu.edu.cn (Z.Z.);; 2 Cancer Stem Cell and Translational Medicine Laboratory, Shengjing Hospital of China Medical University, Shenyang 110022, China; 3 Innovative Cancer Drug Research and Development Engineering Center of Liaoning Province, Shenyang 110022, China; 4 Mount Sinai Center for Translational Medicine and Pharmacology, Icahn School of Medicine at Mount Sinai, New York, NY 10029, USA

**Keywords:** fibrous sheath interacting protein, tumorigenesis, oncogene, breast cancer

## Abstract

Fibrous sheath interacting proteins 1 and 2 (FSIP1 and FSIP2) are evolutionarily conserved testis-specific antigens, exclusively expressed in germ cells of adult human tissues, where they play essential roles in spermatogenesis and testicular development. Aberrant re-expression of FSIP1 and FSIP2, however, has been frequently reported in multiple malignancies, driving oncogenic processes including uncontrolled proliferation, invasion, migration, and metastasis, and correlating with unfavorable clinical outcomes. Their restricted expression in normal tissues, together with their consistent association with poor prognosis across cancer types, highlights their potential as diagnostic biomarkers, therapeutic targets, and prognostic indicators. This review summarizes the structural features and biological functions of the FSIP family, emphasizes recent advances in elucidating their regulatory roles in tumor-associated signaling pathways, and outlines the major challenges and future perspectives in this emerging field.

## Simple Summary

Cancer remains a major health challenge worldwide, and identifying new targets for diagnosis and treatment is crucial. The fibrous sheath interacting protein (FSIP) family, including FSIP1 and FSIP2, are normally found only in the testis, where they support sperm development. However, these proteins reappear abnormally in many cancers, where they promote tumor growth, spread, and resistance to therapies. This review summarizes current knowledge about how FSIPs contribute to cancer progression across multiple cancer types. By understanding their roles, researchers hope to develop new diagnostic tools and targeted therapies that can improve patient outcomes, especially for aggressive and treatment-resistant cancers like triple-negative breast cancer.

## 1. Introduction

Identifying reliable cancer-related genes and uncovering their mechanisms are critical steps in the fight against cancer. The fibrous sheath-interacting protein (FSIP) family, which includes FSIP1 and FSIP2, is part of the cancer/testis antigen (CTA) group [1,2]. In healthy individuals, these proteins are expressed exclusively in male germ cells, contributing to sperm development and motility [3]. However, the FSIPs are re-expressed in various cancers, where they play prominent roles in cancer initiation and progression [4,5,6]. To deepen our understanding of the FSIP family, we provide a comprehensive overview of recent advances in FSIP research related to health and cancer.

The *FSIP1* gene, first identified in 2003, encodes a protein spanning 169 to 1476 base pairs, with a polyadenylation signal between base pairs 1648 and 1653 and a polyA tail beginning at base pair 1670 [3]. The human FSIP1 protein consists of 581 amino acids with a predicted molecular weight of ~66 kDa [3,7]. However, in some Western blot assays it appeared at higher apparent molecular weight. This discrepancy can be explained by: (i) the existence of transcript variants/isoforms; (ii) extensive post-translational modifications (e.g., phosphorylation, glycosylation) that increase apparent size; (iii) possible contributions from expression tags or species-specific sequence differences; and (iv) antibody recognition of higher molecular weight isoforms or protein complexes. These factors collectively account for the larger observed band compared to the theoretical molecular weight. FSIP2, in contrast, is larger at 6781 amino acids, yet binds to A-kinase anchoring protein 4 (AKAP4) through a 122-amino-acid sequence [3,8]. Both FSIP1 and FSIP2 are expressed exclusively in human testes and germ cells, and their expression is regulated tightly in time and space [3,8]. The FSIP family members are not expressed throughout life; instead, their transcription and translation begin with the initiation of spermatogenesis. FSIP1 expression fluctuates in testes between days 8 and 28 post-birth, appearing around day 18, which aligns with the onset of post-meiotic spermatogenesis and suggests the start of FSIP1 transcription with sperm production. As spermatogenesis advances, FSIP1 shifts from the nucleus of round spermatocytes to the anterior region of elongated spermatocytes. In contrast, FSIP2 becomes detectable by day 16, reaching substantial levels by day 18, earlier than both FSIP1 and its binding partner AKAP4, which begins expressing on day 18 [3]. Spermatogenesis is a highly organized process starting with diploid spermatogonium differentiation and culminating in haploid spermatozoa [9,10]. This process consists of three stages: mitotic, meiotic, and spermiogenic [11,12,13,14]. FSIP1 plays a crucial role in spermatogenesis, particularly in acrosomal development and flagellum assembly [8]. In *Fsip1*-deficient (*Fsip1*−/−) mice, there are subtle shifts in the final spermiogenesis stages, resulting in a reduced number of spermatids with abnormal head shapes and incomplete flagella [8]. Normally, mature mouse sperm have elongated heads with mace-like pointed tips. In *Fsip1−/−* mice, the lack of FSIP1 leads to sperm abnormalities, contributing to infertility and highlighting an essential role of FSIP1 in post-meiotic development [8].

FSIP2 is another vital component of the sperm fibrous sheath and is linked to multiple morphological abnormalities of the sperm flagella (MMAF) [15]. Mutations in *FSIP2* can deplete central microtubule structures, such as central pairs, resulting in axonemal defects and MMAF phenotypes characterized by absent, shortened, or coiled flagella [16]. The lack of FSIP2 not only results in these structural abnormalities but also reduces sperm motility and impacts mitochondrial function and fertilization capacity [16,17,18]. Sperm motility depends on dynein, which drives microtubule sliding within axonemes [19,20]; while FSIP1 and FSIP2 are not motor proteins, they play essential roles in sperm function. The fibrous sheath, a critical component of the sperm flagellum, provides a structural framework with longitudinal columns linked by circumferential ribs [3,21,22]. AKAPs, especially AKAP4, are abundant in the fibrous sheath, anchoring protein kinases to specific regions [23]. Research into AKAP-binding proteins identified eight cDNA clones, including *FSIP1* and *FSIP2*, through yeast two-hybrid screening of a mouse testis cDNA library [3].

## 2. Functions of FSIP in Cancers

### 2.1. FSIP in Breast Cancer

Breast cancer is the most common cancer among women and a leading cause of cancer-related mortality worldwide [24,25]. Hormone receptor (HR)-positive breast cancer, characterized by positive expression of estrogen receptor (ER) and progesterone receptor (PR), accounts for over 70% of cases, making them the most prevalent subtype [26,27,28]. A key feature of HR-positive breast cancers is their reliance on intact ER and PR signaling for cell growth and survival, which is dependent predominantly on estradiol [28]. Recent studies have identified high levels of FSIP1 as a poor prognostic factor in HR-positive patients [29,30]. Analysis of data from The Cancer Genome Atlas has confirmed that FSIP1 overexpression is associated with decreased overall survival in these patients [30]. Notably, silencing FSIP1 leads to reduced cell proliferation and migration [5]. In HER2-positive breast cancer, the primary molecular characteristic is the abnormal activation of HER2, a type I receptor tyrosine kinase encoded by the ERBB2 gene, which belongs to the epidermal growth factor receptor family [31]. HER2 overexpression is linked to a more aggressive disease phenotype and poor prognosis [32,33,34]. Studies indicate that FSIP1 expression correlates positively with HER2 levels; clinicopathological analyses reveal that higher FSIP1 levels are associated with increased lymph node metastasis and elevated Ki-67 indices [1]. We have identified FSIP1 as a potential signaling partner of HER2, with experimental evidence suggesting direct binding. Knocking down FSIP1 in HER2-positive breast cancer appears to reduce cell proliferation, enhance apoptosis, induce cell cycle arrest, and attenuate migratory and invasive capacity [1]. FSIP1 promotes the aggressiveness of HER2-positive breast cancer through interactions with the extracellular matrix and by facilitating epithelial–mesenchymal transition, which contributes to enhanced metastasis and may lead to drug resistance [1].

**Figure 1:**
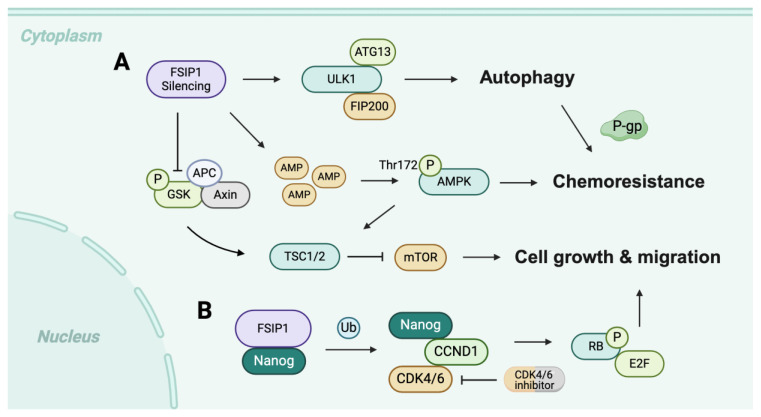
Schematic illustration of the mechanisms by which FSIP1 induces (**A**) cancer cell autophagy, chemoresistance, proliferation, and migration; and (**B**) the potential influence of FSIP1 on the sensitivity of TNBC cells to CDK4/6 inhibitors, possibly through modulation of the Nanog/CCND1/CDK4/6 pathway.

Triple-negative breast cancer (TNBC) poses significant challenges due to its aggressive nature and lack of effective targeted therapies, resulting in a poorer prognosis compared to other subtypes [35]. Current treatment primarily relies on cytotoxic chemotherapy, often combined with immunotherapy for select patients [2,36,37,38,39,40,41,42]. Beyond its role in promoting cell proliferation, FSIP1 is also implicated in drug resistance in TNBC. Docetaxel, which stabilizes microtubules by binding to β-tubulin, shows reduced effectiveness in FSIP1-deficient TNBC cells, suggesting that microtubule stability is compromised in the absence of FSIP1 [43,44]. FSIP1 also inhibits autophagy, which is essential for maintaining cellular homeostasis [2,45]. While docetaxel is ineffective against TNBC cells lacking FSIP1, co-treatment with autophagy inhibitors, such as 3-methyladenine or bafilomycin A1 restores sensitivity. AMP-activated protein kinase (AMPK), a key stress sensor, inhibits mTOR signaling, and enhances autophagy [46,47]. Although silencing FSIP1 does not alter total mTOR and AMPK expression, it enhances AMPK activation through increased phosphorylation and reduces mTOR signaling via decreased phosphorylation of mTOR [47]. Collectively, FSIP1 silencing decreases oxygen consumption rates and mitochondrial activity, contributing to reduced sensitivity of TNBC cells to chemotherapy (Figure 1A). Furthermore, recent studies show that, in addition to inhibiting autophagy, FSIP1 can enhance resistance to chemotherapeutic drugs by stabilizing MRP1 [7]. Increased levels of apoptotic proteins, such as Bax, cleaved caspase 3, and cleaved PARP in FSIP1 knockout cells further support its anti-apoptotic function. Our studies showed that FSIP1 may influence the sensitivity of TNBC cells to CDK4/6 inhibitors, potentially through modulation of the Nanog/CCND1/CDK4/6 pathway (Figure 1B) [48]. FSIP1 may interacts with Nanog, stabilizing it by preventing ubiquitination and subsequent degradation. Thus, in FSIP1-deficient cells, reduced expression of CCND1 and CDK4/6 leads to decreased RB1 phosphorylation and potential cell cycle arrest, significantly diminishing sensitivity to CDK4/6 inhibitors and highlighting FSIP1 as a potential predictive marker for TNBC responsiveness to these drugs.

Patients with TNBC experience poorer outcomes compared to those with other breast cancer subtypes [49,50,51]. With no targeted therapies available, conventional chemotherapy remains the standard of care [52]. Identifying novel biomarkers for TNBC could facilitate the development of more effective therapies. The absence of reliable biomarkers for chemotherapy response or resistance has hindered the advancement of new treatments for TNBC. Currently, while TNBC lacks predictive and prognostic markers, FSIP1 emerges as a significant indicator of chemotherapy resistance and is closely linked to patient prognosis [53]. Consequently, FSIP1 holds potential as a reliable marker for predicting poor outcomes and drug resistance in TNBC, positioning it as a promising therapeutic target for this challenging subtype.

### 2.2. FSIP in Testicular Germ Cell Tumor (TGCT)

The primary histological classifications of TGCTs consist of seminoma, which mirrors undifferentiated germ cells, and non-seminoma, characterized by varying levels of differentiation [54,55,56]. FSIP2 was identified as the top amplification gene based on copy number variation analysis in TGCT [57]. FSIP2 is notably expressed in germ cells and is believed to serve as a linking protein, anchoring AKAP4 to the fibrous sheath [57]. Disruptions in the development of the fibrous sheath and mutations in AKAP4 have been linked to male infertility, a known risk factor for TGCT.

### 2.3. FSIP in Urological Cancer

Bladder cancer is the most common malignancy in urinary system, with increasing incidence and recurrence rates [58]. Single nucleotide polymorphisms in the FSIP1 gene are strongly linked to the risk of arsenic-related bladder cancer, with variants significantly increasing susceptibility in populations exposed to high arsenic levels but showing no association in those with low exposure [59]. FSIP1 is significantly upregulated in advanced bladder cancer with lymph node metastasis, and multivariate Cox regression analyses indicate its potential as an independent prognostic indicator for the disease [60]. Clear cell renal cell carcinoma (ccRCC) is the most common pathological type of RCC and accounts for the majority of RCC-related deaths. FSIP2 expression is significantly elevated in ccRCC tissues compared to surrounding normal tissues and is associated with abnormal platelet counts, distant metastasis, and reduced survival in ccRCC patients [61].

### 2.4. FSIP in Gastrointestinal Cancers

Gastric cancer (GC), the most common malignant tumor of the digestive system, is characterized by a poor survival rate, particularly in advanced stages, highlighting the ongoing challenge of developing effective targeted treatments [62]. Further research in identifying novel oncogenic genes and molecular mechanisms associated with GC is crucial for improving the prognosis of patients. FSIP1 levels are elevated in GC tissues compared to normal tissues and are associated with advanced pathological stages, nervous system involvement, and poorer outcomes, including shorter disease-specific and progression-free survival [63]. Multivariate analysis identifies FSIP1 as an independent prognostic risk factor, with a prediction model incorporating FSIP1 expression, N classification, and T classification being established to further highlight the adverse impact of high FSIP1 expression on patient outcomes [63]. Notably, it was found that *FSIP1* knockdown could inhibit the migration and invasion of GC cells by downregulating EMT-related markers, such as N-cadherin and vimentin.

Colorectal cancer (CRC) is a significant global health concern, with approximately 700,000 new cases of rectal cancer diagnosed worldwide each year [64,65,66]. FSIP1 was found to localize primarily in the cytoplasm of CRC tumor cells, and its expression in CRC tissues was significantly higher than that in adjacent tissues. High expression of FSIP1 was strongly correlate with T stage, N stage, and histological stage, and poor survival [4]. Additionally, FSIP2 was originally characterized as an oncogene in esophageal squamous cell carcinoma (ESCC), as elevated expression of FSIP2 indicates a poor prognosis for ESCC patients. Furthermore, the elevated expression of FSIP2 in ESCC also associated with gross type, lymphatic vascular invasion, and T stage [67].

### 2.5. FSIP in Oral Mucosal Melanoma (OMM)

OMM arises from the transformation and clonal expansion of melanocytes, most commonly occurring on the hard palate and gingiva, and represents approximately 2% of all mucosal and cutaneous melanomas [68]. Despite treatment options such as surgery, with or without adjuvant radiotherapy, the prognosis remains poor, with survival rates of 43.4% at 3 years, 33.1% at 5 years, and 15.4% at 10 years, while targeted therapies remain underutilized [69]. Notably, OMM exhibited a significantly lower frequency of loss-of-function (LOF) mutations and an upregulation of the *FSIP1* gene compared to non-oral mucosal melanomas [70]. Since LOF mutations can mimic the effects of gene knockdown, the relative scarcity of such mutations in OMM suggests a potential upregulation or activation of FSIP1, which may contribute to OMM progression.

### 2.6. FSIP in Skin Cutaneous Melanoma (SKCM)

Cutaneous melanoma, commonly referred to as SKCM, is a prevalent form of skin cancer originating from unregulated proliferation of epidermal melanocytes [71]. FSIP2 mutations discerned within clinical treatment cohorts predominantly comprised missense mutations, which consequentially attenuate the innate function of FSIP2 [72]. FSIP2 mutations are associated with elevated tumor mutational burden and neoantigen load, both of which are strongly linked to the effectiveness of immunotherapies. Additionally, samples with FSIP2 mutations exhibit reduced copy number variations and lower levels of regulatory T cells. [72]. The underlying mechanism is likely that the FSIP2 mutation has the potential to decrease AKAP4 expression and thereby impair the linkage between PKA and AKAP4 [72]. This disruption subsequently influences the PKAI-mediated anti-tumor immune suppression triggered by regulatory T cells. Furthermore, the FSIP2 mutation manifested pronounced downregulation in pathways pertinent to tumor advancement (such as MAPK and FGFR), immune modulation, and IL-2 synthesis.

### 2.7. FSIP in Non-Small Cell Lung Cancer (NSCLC)

It has been shown that the expression levels of FSIP1 mRNA and protein was increased in NSCLC and corresponding adjacent non-tumor tissues [73]. Survival analysis revealed that patients in the FSIP1-positive cohort had a significantly lower 5-year overall survival rate compared to those in the FSIP1-negative cohort (35.4% vs. 56.3%) [73]. Moreover, positive FSIP1 expression was associated with a more advanced TNM stage. Notably, the concordance index for TNM staging combined with FSIP1 status exceeded that of TNM staging alone, suggesting that elevated FSIP1 expression may serve as an independent predictor of poor prognosis in NSCLC, thereby enhancing prognostic capability.

## 3. Opportunities and Challenges

The exploration of FSIP1 and FSIP2 in various cancers highlights both their potential and the hurdles yet to be overcome (Table 1). As CTAs, FSIP1 and FSIP2 exhibit unique roles in cancer biology. FSIP1 is critical for tumor cell division, where its absence disrupts mitotic processes and enhances paclitaxel sensitivity, while its overexpression induces mitotic errors. Despite these insights, current research predominantly focuses on breast cancer, where FSIP1 is highly expressed and associated with poor prognosis. The breast tumor microenvironment and the role of FSIP1 in immune infiltration and drug resistance, especially in TNBC, underscore the need for targeted therapeutic strategies. According to some research, inhibition of FSIP1 expression in HER2-positive breast cancer cells results in reduced cell proliferation, increased apoptosis, and attenuated cell migration and invasiveness. While the mechanisms of FSIP1 in HER2-positive breast cancer and its interaction with MRP1 have been explored, its role in TNBC resistance and autophagy inhibition remains under investigation. FSIP2 plays an essential role in spermatogenesis by anchoring AKAP4 to the fibrous sheath, and genetic variants have been linked to multiple morphological abnormalities of the sperm flagella and infertility. In cancer, FSIP2 alterations or overexpression have been reported across several contexts, including testicular germ cell tumors, clear cell renal cell carcinoma, esophageal squamous cell carcinoma, and cutaneous melanoma, where it is variably associated with tumor progression, metastasis, poor prognosis, or immunotherapy response. However, compared with FSIP1, studies on FSIP2 remain relatively sparse. Current findings are largely based on limited genomic or clinical datasets, and mechanistic insights into how FSIP2 contributes to cancer progression are still lacking.

Post-translational modifications play crucial roles in regulating the stability and function of FSIPs, thereby influencing sperm tail assembly and motility. Phosphorylation has been shown to modulate the interaction between FSIP1 and key structural proteins such as AKAP3/4, with dynamic changes in phosphorylation, particularly within the C-terminal region of FSIP1,potentially driving the maturation of the fibrous sheath [3]. In addition to phosphorylation, ubiquitination serves as a critical quality-control mechanism for FSIPs. Elevated ubiquitination levels observed in sperm from patients with dysplasia of the fibrous sheath suggest that ubiquitination helps eliminate aberrant FSIPs, preventing structural defects and motility impairment [74]. Moreover, ubiquitination appears to operate through dual mechanisms: K48-linked ubiquitin chains directing proteasomal degradation, and K63-linked chains facilitating autophagy-mediated clearance, thus ensuring precise temporal regulation of FSIP turnover during spermatogenesis and fertilization [75]. Collectively, these findings highlight that FSIP function is tightly regulated by phosphorylation and ubiquitination, which together orchestrate fibrous sheath assembly, maintenance, and degradation. Further investigations are needed to elucidate the functional roles of post-translational modifications in FSIP function.

Besides post-translational modifications, further investigation is needed into the roles of promoters, such as proteins, transcription factors, and hormones that regulate FSIP1, in FSIP-mediated cancer regulation. Based on promoter architecture and analogies with regulatory patterns of related genes, it is plausible that FSIP1 transcription is modulated through multiple layers of control. The promoter region may harbor SP1-binding sites [76], whose activity could be enhanced under HER2/PI3K/AKT signaling, while NF-κB response elements may contribute to FSIP1 induction in inflammation-associated contexts [77]. Likewise, EMT-related transcription factors such as Snail and Twist could potentially interact with E-box motifs under TGF-β stimulation to promote FSIP1 upregulation [6]. In addition, epigenetic modifications, including promoter CpG hypomethylation and activating histone marks such as H3K4me3, may facilitate chromatin accessibility and transcription factor recruitment [78]. Furthermore, noncoding RNAs, for instance, lncRNAs interacting with promoter regions or miRNAs indirectly modulating transcription factors such as SP1, may add another regulatory layer [76]. Although these mechanisms remain speculative in the absence of direct experimental validation, they provide a rational framework for understanding how FSIP promoter regulation could underlie its aberrant overexpression and oncogenic functions in cancer progression.

FSIP1 and FSIP2, as CTAs, suggest evolutionary links between testis-specific protein expression and cancer development, and represent promising therapeutic targets. Key opportunities and challenges in targeting FSIPs are summarized below:

1. CTA-based immunotherapy:

- Exploits tumor-restricted expression for selective targeting (e.g., cancer vaccines, TCR-engineered T cells, adoptive cell transfer).
- Requires clear evidence of antigen processing and presentation in tumors and sufficient epitope immunogenicity.
- Immune privilege of testes may reduce systemic toxicity, but potential effects on spermatogenesis need careful assessment.

2. Antibody-based treatment strategies:

- First, determine whether FSIPs or stable extracellular fragments are accessible on the tumor surface.
- If predominantly intracellular, conventional antibodies are limited; alternative approaches such as TCR-mimic antibodies or bispecific T cell engagers may be required.
- Success depends on robust validation of antigen presentation across patient tumors.

3. Targeted protein degradation (PROTACs/molecular glues):

- Provides a route to pharmacologically target intracellular FSIPs.
- Feasibility depends on identifying small-molecule ligands with adequate affinity and selectivity, which is challenging given the protein size and complexity.
- Structure-guided, peptide-based, or degron-mimetic strategies may serve as starting points for ligand discovery.

## 4. Conclusions

FSIP1 and FSIP2 have emerged as promising targets for cancer diagnosis, prognosis, and therapy, yet their full therapeutic potential remains largely untapped. Mechanistic studies indicate that FSIP1 may interact with HER2 to modulate downstream signaling pathways and potentially influence processes such as autophagy and the Nanog/CCND1/CDK4/6 axis. However, these observations are primarily derived from a limited number of in vitro and in vivo models. Consequently, the reported associations should be interpreted cautiously, as they do not yet establish definitive causality. Validation in independent cohorts and clinical settings is essential to substantiate these findings.

Continued investigation into the molecular mechanisms and functional roles of FSIPs will be critical for translating these insights into effective cancer therapies. FSIP1 and FSIP2 present multiple avenues for therapeutic intervention, including immunotherapy and targeted protein degradation strategies. Advancing these cancer-testis antigen (CTA) targets toward clinical application will require rigorous antigen validation, precise epitope mapping, modality-specific optimization, and comprehensive safety assessments. Given the heterogeneity of tumors and the variability of patient-specific expression, biomarker-guided patient selection and systematic preclinical evaluation are crucial to ensure both efficacy and safety in FSIP-targeted treatments. Overall, FSIPs represent a compelling yet complex frontier in cancer therapy, with significant potential contingent on careful mechanistic and translational research.

## Figures and Tables

**Table 1:** Summary of FSIP1 and FSIP2 expression, correlations, and prognostic implications across cancer types.

Cancer Type	FSIP1 Expression	FSIP2 Expression	Clinical Correlations	Prognostic Implications
Breast cancer	Overexpressed in multiple subtypes; correlates with ER/PR status and HER2 levels	–	Promotes proliferation, migration, EMT, autophagy inhibition, drug resistance; interacts with HER2; may stabilize Nanog/CDK4/6	High FSIP1 linked to poor survival; predictive marker for drug resistance and CDK4/6 inhibitor sensitivity
Testicular germ cell tumor	–	High copy number amplification in TGCT	Anchors AKAP4 to fibrous sheath; associated with germ cell biology	Potential oncogenic role; limited prognostic data
Bladder cancer	Upregulated in advanced stages with lymph node metastasis	–	SNP variants linked to arsenic-related susceptibility	High FSIP1 = independent predictor of poor prognosis
Clear cell renal cell carcinoma	–	Elevated compared to normal tissue	Associated with abnormal platelet count, distant metastasis	High FSIP2 expression linked to reduced survival
Gastric cancer	Elevated in tumor vs. normal tissue	–	Correlates with advanced stage, nervous system invasion, EMT marker expression	High FSIP1 = poor disease-specific and progression-free survival
Colorectal cancer	Strong cytoplasmic expression in tumor vs. normal	–	Correlates with T stage, N stage, histological stage	High FSIP1 = poor overall survival
Esophageal squamous cell carcinoma	–	Elevated in tumor	Associated with gross type, lymphatic vascular invasion, T stage	High FSIP2 = poor prognosis
Oral mucosal melanoma	Upregulated compared to other subtypes	–	Relative scarcity of LOF mutations suggests upregulation of FSIP1	High FSIP1 may contribute to OMM progression
Skin cutaneous melanoma	–	FSIP2 mutations detected	Linked to reduced Treg infiltration, high tumor mutational burden, altered MAPK/FGFR pathways	FSIP2 mutation associated with improved immunotherapy responsiveness
Non-small cell lung cancer	Overexpressed vs. adjacent tissue	–	Correlates with advanced TNM stage	High FSIP1 = poor 5-year survival

## Data Availability

Not applicable.

## Abbreviations

AKAP4	A-kinase anchoring protein 4
AMP	Adenosine monophosphate
AMPK	AMP-activated protein kinase
CCND1	Cyclin D1
CDK2	Cyclin-dependent kinase 2
CDK4	Cyclin-dependent kinase 4
CRC	Colorectal cancer
CTA	Cancer/testis antigen
DUSP1	Dual-specificity protein phosphatase 1
EMT	Epithelial–mesenchymal transition
ER	Estrogen receptor
ERBB2	Erb-B2 receptor tyrosine kinase 2
ESCC	Esophageal squamous cell carcinoma
FGFR	Fibroblast growth factor receptor
FSIP	Fibrous sheath-interacting protein
GC	Gastric cancer
HER2	Human epidermal growth factor receptor 2
HR	Hormone receptor
IL	Interleukin
MAPK	Mitogen-activated protein kinase
MMAF	Multiple morphological abnormalities of the sperm flagella
MRP1	Multidrug resistance-associated protein 1
NSCLC	Non-small cell lung cancer
OMM	Oral mucosal melanoma
PARP	Poly (ADP-ribose) polymerase
PCBP2	Poly(rC)-binding protein 2
PKAI	Protein kinase A type I
PR	Progesterone receptor
RB1	Retinoblastoma protein 1
RCC	Renal cell carcinoma
SKCM	Skin cutaneous melanoma
